# A Comprehensive Review on the Applications of Exosomes and Liposomes in Regenerative Medicine and Tissue Engineering

**DOI:** 10.3390/polym13152529

**Published:** 2021-07-30

**Authors:** Mojtaba Shafiei, Mohamed Nainar Mohamed Ansari, Saiful Izwan Abd Razak, Muhammad Umar Aslam Khan

**Affiliations:** 1 Bioinspired Device and Tissue Engineering Research Group, School of Biomedical Engineering and Health Sciences, Faculty of Engineering, Universiti Teknologi Malaysia, Skudai 81300, Johor, Malaysia; mojtabi.shafiei@gmail.com (M.S.); umaraslam@utm.my (M.U.A.K.); 2 Institute of Power Engineering, Universiti Tenaga Nasional, Kajang 43000, Selangor, Malaysia

**Keywords:** exosome, liposome, scaffolds, stem cell, drug delivery, tissue engineering, regenerative medicine

## Abstract

Tissue engineering and regenerative medicine are generally concerned with reconstructing cells, tissues, or organs to restore typical biological characteristics. Liposomes are round vesicles with a hydrophilic center and bilayers of amphiphiles which are the most influential family of nanomedicine. Liposomes have extensive research, engineering, and medicine uses, particularly in a drug delivery system, genes, and vaccines for treatments. Exosomes are extracellular vesicles (EVs) that carry various biomolecular cargos such as miRNA, mRNA, DNA, and proteins. As exosomal cargo changes with adjustments in parent cells and position, research of exosomal cargo constituents provides a rare chance for sicknesses prognosis and care. Exosomes have a more substantial degree of bioactivity and immunogenicity than liposomes as they are distinctly chiefly formed by cells, which improves their steadiness in the bloodstream, and enhances their absorption potential and medicinal effectiveness in vitro and in vivo. In this review, the crucial challenges of exosome and liposome science and their functions in disease improvement and therapeutic applications in tissue engineering and regenerative medicine strategies are prominently highlighted.

## 1. Introduction

**Figure 1:**
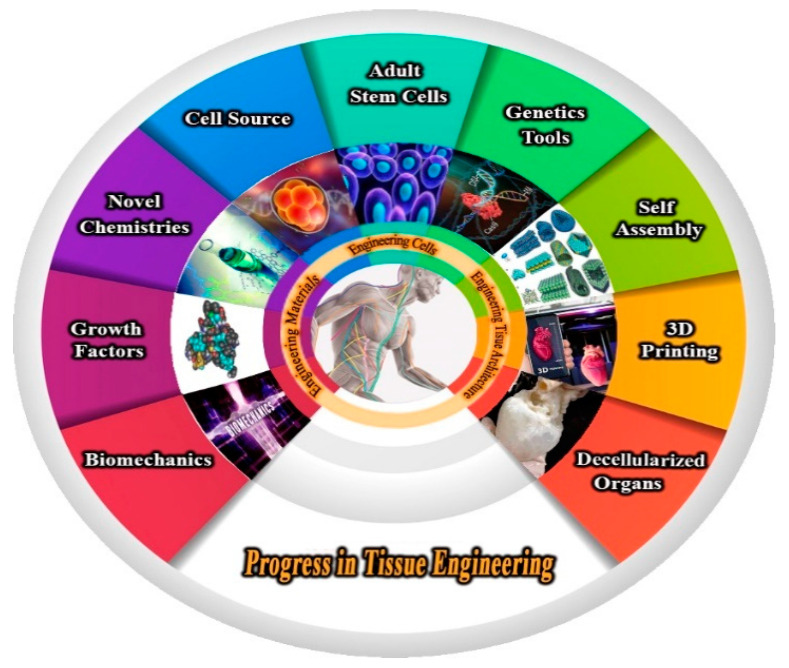
Schematic representation of Summary Tremendous progress has been achieved in tissue engineering and regenerative medicine in the past decade.

Tissue engineering is a biomedical engineering specialty that focuses on cell types, biologically compatible substances, and appropriate the metabolic (e.g., cytokines such as growth factor), the physical, and mechanical factors (e.g., mechanical loading by cyclic) to rebuild, combine, sustain, strengthen, or substitute tissue-like structures [1]. Tissue engineering and regenerative medicines are substantially elementally speedy, typically growing processes to create real, novel limbs and types of tissues in the bodies. Under other circumstances, it is a discipline that primarily aims to replace or improve the biomedical role of the organ, or even fundamental tissues, by modifying cells through the outer membrane environment among various circumstances [2,3]. The field of tissue engineering and regenerative medicine (TERM) has become a comparatively modern discipline that originated in the earlier twentieth century. This is composed of basic sciences such as biology, stem cell, advanced functional materials, and scaffold fabrication technologies, as well as the newest additive manufacturing (AM) (generally referred to as three-dimensional (3D) printing), to realize functional tissue/organ repair or reconstruction [4]. The world of tissue engineering has made considerable strides in the last decade to solve enormous obstacles. The shortage of sustainable sources of functional cells, a lack of suitable biomaterials, and the failure to manufacture large, vascularized tissues were among the limitations that were overcome using material science, chemistry, engineering methods, and the convergence of these disciplines (Figure 1) [5]. As evidenced by the rapid increase in the number of people on the waiting list to the donor list, the medical need for tissue engineering and completely regenerative medicine has significantly increased. However, the donors’ incompatibility with the recipients’ tissues, transportation issues, and limited time for transplantation must be remembered.

### 1.1. The Current State and Background of Tissue Engineering and Regenerative Medicine

After a long elementary period over the past three decades, the current state of TE has largely seen continuous evolution. It has had visible expert, experience, and technological improvements from associated fields, such as materials science and engineering, rapid prototyping (RP), nanoscience and nanotechnology, cellular biology, and cell and developmental biology [1]. In general, the special advances that tissue engineering has benefited from in recent years are remarkable. These include gene-editing technology, including CRISPR (Clustered Regularly Interspaced Short Palindromic Repeats) [6,7]; stem cell technology [8,9], which includes caused pluripotent stem cells (iPSCs) [10]; three-dimensional (3D) bioprinting technology [11,12]; integration of nanotechnology [13,14]; and novel biomaterials [15,16]. A historical perspective may begin with ancient civilizations;it is explicitly believed that the earliest gold dental prosthesis was built in Egypt, around 2500 BC [17,18]. In the historical Egyptian town of Thebes, Nerlich and collaborators found a large artificial toe of the foot, which is believed to be the oldest known functioning prosthesis (950–710 BC) [19]. During 1546–99, Gaspare Tagliacozzi Bologna, an Italian surgeon, was the first to initiate a nose prosthetic that he had constructed, and write a book on plastic surgical procedures involving the restoration of the nose and reconstruction of the ear flap [20]. Due to the fact that the system of medicine, surgery, the science of infection prevention, the development in anesthesia rapidly advanced in the nineteenth century, this development has allowed the primary systems of living tissues and organs to function better [21]. In the mid-to-late nineteenth century, skin grafts were the primary tissue-based therapies; skin allografts were generally used in conditions wherein there is substantial skin loss, and the creation of strategies to hold cells and tissues enabled allograft skin banking [22,23,24]. Shortly after, the first successful complete renal transplantation of fundamentally monogamous identical twins was conducted, which reduced the risk of kidney rejection. This recognizes the unique bond between the donor and the recipient, reducing the adverse effects of immunosuppressive therapy [25,26]. According to previous research, in recent years, regenerative medicine has made promising progress towards healing or replacing tissues, which is expected to be possible to utilize in the near future, in place of traditional therapies which cause significant side effects [27]. Successful research studies have been carried out in the past few years, and massive improvement and development had been described regarding the reconstruction of diverse human tissue replacement and prosthesis, including the uterus [28], adipose tissue [29,30], cardiac [31], blood vessels [32,33], lung [34,35], kidney [36,37], skin [38,39,40], trachea [41,42], intestine [43], cartilage [44,45], bladder [46,47], dental [48,49], cornea [50,51], nerve [52,53], and bone [54,55,56,57].

### 1.2. Biology of Exosomes and Liposomes

#### 1.2.1. Liposomes

**Figure 2:**
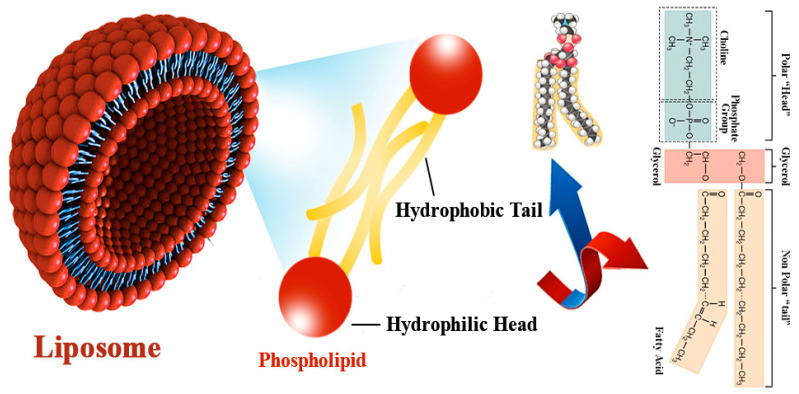
Schematic of a liposome. Liposomes are circular vesicles with internal aqueous hearts primarily covered by one or predominantly more layers of phospholipids. These layers are generally principally amphiphilic substances, with double surely lipophilic substance tails and a generally polar head (hydrophilic).

The name liposome is derived from Greek words: ‘Lipos,’ which means fat, and ‘Soma,’ which means body. In most cases, self-assembled vesicles can encapsulate aqueous solutions and hydrophobic compounds [58]. Liposomes have been discovered by Alec D Bangham, who observed the unique behaviors of lipids by transmission electron microscope (TEM), consisting of single or multiple concentric lipid bilayers encapsulating an aqueous compartment (Figure 2) in the 1960s at the Babraham Institute, University of Cambridge [59,60]. Since their discovery, liposomes have been utilized as a model for many studies to understand their biophysical and biochemical properties and possible applications [61]. They have been used as model membrane systems to examine the primary nature of cell membranes [62], as drug delivery systems in pharmacology [63,64,65], in biochemistry [62,66,67], diagnostics [68], in imaging [69,70], molecular biology [71], food technology and cosmetic industries [72,73,74], microfluidic technologies [75,76], in analytical methods [67], as a template for the production of nanogels [77], as the Biolubricants carrier [78,79,80], and in tissue engineering and regenerative medicine [60,81,82].

##### Major Structural Components of Liposomes

Liposome membranes are composed of lipid layers, together called a bilayer membrane. Size of liposomes can vary in an extensive range of 50–1000 nm, and they mostly serve as convenient transport vehicles [83].

##### Phospholipids

Phospholipids are the main substantially structural component of the liposomes’ membrane, and various styles of phospholipids exist. Examples of phospholipids are: (1) Phosphatidylcholine (lecithin) (pc), (2) Phosphatidylethanolamine (cephalin) (PE), (3) Phosphatidylserine (ps), (4) Phosphatidylinositol (PI), and (5) Phosphatidyl glycerol (PG) [58].

##### Cholesterol

The cholesterol molecules inside the membrane increase separation among choline head groups, which significantly reduces the normal hydrogen bonding and electrostatic interaction, with very excessive concentrations of up to 1:1 or 2:1 molar ratio of cholesterol phosphatidylcholine [58].

##### Classification of Liposomes

Based on the size of liposomes and the number of bilayers, liposomes are specially classified into two categories [84,85]: (1) multilamellar vesicles (MLVs) and (2) unilamellar vesicles.

The size of the vesicles is an essential factor affecting half of the lifestyles of liposomes in circulation. In summary, liposomes generally may be classified into the following kinds using their sizes, lamellarity, and different electromagnetic charging, as shown in Table 1 [84,86,87].

#### 1.2.2. Exosomes

##### The Discovery of Exosomes

**Figure 3:**
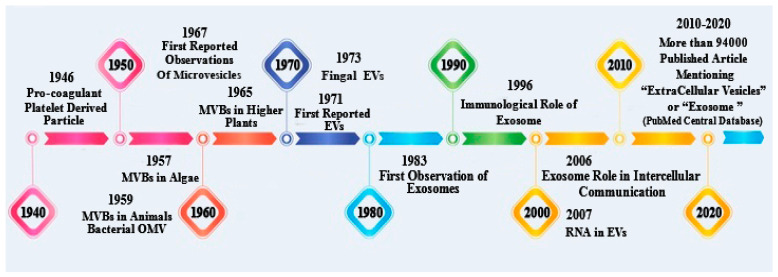
The timeline illustrates key discoveries in the history of extracellular vesicle (EV) discovery and research.

It is mainly acknowledged that extracellular vesicles (EVs) are membrane-contained vesicles released in an evolutionarily conserved manner by all cells of prokaryotes and eukaryotes. Exosomes had been recognized using Pan and Johnstone (1983); EVs can be significantly divided into two categories, ectosomes (length range of ~50 nm to 1 mm) and exosomes (length range of ~40 to 160 nm) [88,89]. Extracellular vesicles (EVs) probably play the most crucial role in different cell membrane signaling mechanisms. Following the discovery of extracellular vesicles, and research into their biogenesis, it was found that endosomes are their primary source. To summarize, observation of extracellular vesicles (EVs) shows that the difference in exosomes’ final content material relates to subsequent interactions with distinct intracellular vesicles and organelles [89]. Presently, extracellular vesicles (EVs) play a role in various cell-to-cell communication pathways (by transporting messenger RNA (mRNA), microRNA (miRNA), and protein synthesis), as seen in the timeline (Figure 3) [90,91,92,93]. Additionally, this timeline demonstrates that MVBs were first recognized in the 1950s, in photosynthetic eukaryotic organisms consisting of algae and animal cells, while bacteria were found to have external membrane vesicles (OMVs) [91]. MVBs were first discovered in fundamentally plant species about ten years after algae were discovered; EVs were found in fungi, once again, almost ten years later, in 1973. Exosomes were first literally reported in 1983, and the function of B lymphocyte-secreted exosomes in the protective immune system was first discovered after another ten years, in 1996 [90,92]. Later, in 2006, the role of exosomes in the transport of RNA and protein between cells was remarkably addressed. In 2007, EVs were discovered to bear messenger RNA (mRNA) and small non-coding RNA (sncRNA), allowing genetic information to be shared by cells. In total, there are nearly 94,000 clinical studies in the database that use the term “extracellular vesicles” such as exosomes, which have led to a wide range of innovative studies in the central PubMed database [90,91]. Figure 2 depicts a timeline of the first discovery of extracellular vesicles (EVs) and some of the most significant breakthroughs in the field of EVs research.

##### Mechanisms of Exosome Formation and Biogenesis

Processes linked to exosome genetic recombination, biogenesis, and release were all regulated by a complex set of mechanisms [94]. A potential role of constitutive or inducible secretion in exosomes occurs on normal physiological conditions through the complex set of underlying mechanisms [95]. Depending upon the cell’s state, one or both of these pathways can be significantly operational. A potential role of constitutive secretion on exosomes happens distinctly in a variety of cell types in broadly standard physiological and pathological settings. Rab (family) proteins are small guanosine triphosphatases (GTPases), which contribute significantly to exosome release [96]. Furthermore, the number of essential intracellular compartments are implicated with exosome biosynthesis and dissemination, including; the functional multivesicular bodies (MVBs), the intraluminal vesicles (ILVs), and the Golgi apparatus. The endosomal sorting complexes required for transport (ESCRT) machinery is made up of cytosolic protein complexes [97,98].

##### Composition of Exosomes

**Figure 4:**
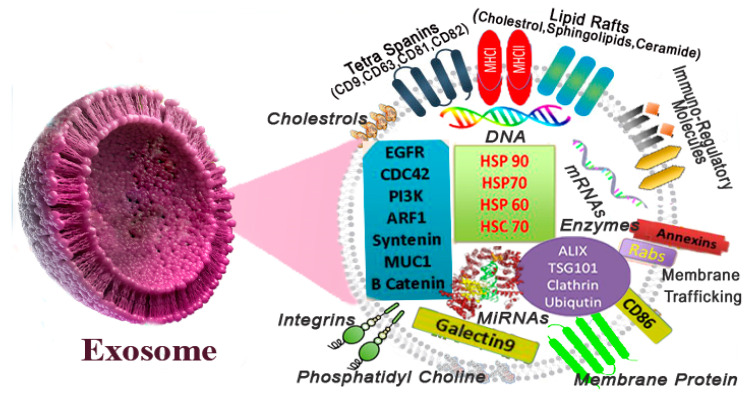
Schematic showing Exosome composition and structural elements.

Exosomes certainly incorporate a complicated combination of numerous proteins, lipid molecules, different nucleic acid species, and other metabolites molecules. However, exosomes do not contain cellular organelles such as the nucleus, endoplasmic reticulum (ER), mitochondria, and the Golgi, and are not made up of a random collection of proteins, as depicted in Figure 4 [99]. ExoCarta is a web-based compendium of exosome proteins, RNA, and lipid database information (http://www.exocarta.org since in 17 April 2009), curated over the past few years and continuously updated [100]. Current research has proven that exosomes include approximately 4563 proteins, 194 lipids (eight categories), 1639 messenger ribonucleic acids (mRNAs), and 764 micro RNAs (miRNAs). Lipids are the major components of exosomes, and exosome-specific protein conformation can also be obedient to the cell kind or tissue birthplace from which it originates [101].

#### 1.2.3. The Exosomes and Liposomes Similarities and Differences

Exosomes and liposomes with identical physicochemical properties are vesicular structures, composed of one lipid bilayer, with mean diameters ranging from 50 nm to 120 nm. Liposomes are eventually susceptible to enzymatic degradation or phagocytic attack, as the liposomes are artificial and do not have the biological markers to deceive white blood cells [102,103,104]. The critical distinction between liposomes and exosomes is the complicated surface structure of exosomes and, with greater specificity, membrane proteins such as tetraspanins. Liposomes, on the other hand, typically do not have proteins in or on their lipid bilayers. Exosomal proteins are needed for proper targeting and uptake by recipient cells [102]. Exosomes have also been widely studied as transportation motors due to their intrinsic characteristics such as flexibility, poor immunogenicity, and potential to pass biological boundaries, as well as their excellent biocompatibility. Exosomes will transfer signaling molecules, accompanied by microRNAs (miRNAs), messenger RNA (mRNA), fatty acids, and proteins. However, in contrast to suitable liposomes, exosomes cannot presently be used to deliver exogenous hydrophilic macromolecules efficaciously. Due to their limited scales and sizes, they may evade phagocytosis function, increasing and supplying the freight in circulation [102,105].

#### 1.2.4. Engineered Extracellular Vesicles by Way of Membrane Fusion with Synthetic Lipids

The use of the well-known liposome engineering technology to engineer exosomes has been advised to remedy their limits due to the numerous biochemical resemblances amongst conventional liposomes and natural exosomes [102]. According to the reports, positive interaction between the two fields can be promoted due to the research [106]. Exosome superiority in unusual lipid compositions may help with cellular internalization, circulation or storage targeting, advanced liposomal delivery, higher efficiency drug delivery, and biocompatibility [102,107]. The investigation’s final findings revealed that the CRISPRCas9 system was successfully transported to MSCs using hybrid exosomes obtained by simple liposome incubation [108].

#### 1.2.5. Scaffold Techniques and Designs Based on Liposome and Exosome in Tissue Engineering

At present, synthetic scaffolds have been used as a support system for cell cultures and cell growth superiority in reconstructing damaged tissues or organs [109]. The scaffold temporarily aids in the cell-for-cell regeneration duration, and biodegrades gradually both inside and during the recovery period, resulting in an entirely new tissue with the same structure and properties [110]. The scaffold’s degradability property negates any need to remove the substance later, and thereby prevents the side effects of foreign materials left in the body. Therefore, to accomplish cell diffusion and 3D tissue forming, the used scaffold should also meet the precise chemical, unique mechanical, and distinct physical requirements [109]. Scaffolds usually have unique characteristics, i.e., excessive porosity, acceptable microporous extent, hemocompatibility, and desirable biodegradable rate [111]. The extracellular matrix (ECM) has obtained significant interest amongst researchers regarding the manufacture of scaffolds due to its excessive biological compatibility, biological degradability, and the opportunity for rapid in vivo remodeling [112]. Different materials, including metallic material, significantly advanced ceramics, polymeric materials, composites, and hydrogels [113], were over-researched to manufacture scaffolds in the last two centuries [114,115]. A range of particular techniques has been explored in this field, including top-down/bottom-up approaches by using beneficial cell sheets [116], the layer-by-layer (LbL) cell assembled surfaces technique [117], 3D printing [118], ceramic process [119], electrospinning [120], and biodegradable polymeric scaffolds [121].

#### 1.2.6. Combining Scaffolds with Liposomes

Generally, while advanced liposome-loaded composite systems are used for current tissue and organ engineering applications in biomedicine, non-modified scaffolds display a limited capability to promote tissue regeneration and remedy diseases [60,122]. Scaffolds are presently extensively used in specific organs for identification, fault diagnosis, communication, reconstruction, and tissue repair of function. Essentially, there are primarily three kinds of scaffolding: metallic scaffolds, which include safe alkali-metal-based stable scaffolds, and a few as the primary alloy scaffolds surface; inorganic composite scaffolds, including hydroxyapatite and bioactive glass(BGs) strong; and natural organic scaffolds, e.g., scaffolds made of polymeric materials [60]. Scaffolds may equip the positional defect area with the required mechanical support. They can regulate blood and body fluid circulation, and these materials supply a sufficient and cell proliferation-friendly microenvironment. However, the fabrication of drug-loaded porous scaffolds is essential to broaden the potential of scaffolds’ clinical applications, as biodegradable scaffolds themselves have minor medicinal effects in the local area. Therefore, they do not satisfy different clinical requirements. When paired with biomaterial scaffolds, the chiefly therapeutic ability of liposomes can be reinforced. Liposomes benefit from extended physical and mechanical strength, favorable rheological characteristics, and the natural environment offered by scaffolds for increased tissue formation while allowing, in addition to further functionalization, the biocompatible transport of hydrophilic and lipophilic substances to arrive at targeted delivery [123]. Swiss Cilag submitted a patent in 1988 for the first liposomal medicine product (econazole in a liposomal gel) to treat pores and uncommon skin diseases, which was once the most popular liposome biosensor-based product and is currently accessible in Europe and other worldwide places [124]. The liposome configuration is quite sensitive to organic compounds, pH, and temperature, therefore many different ways have been suggested to immobilize liposomes on the surface of the scaffold [125,126,127,128]. On the surface of the scaffold, there are two approaches to immobilize liposomes: (i) non-precise immobilization, meaning the liposomes are adsorbed on the surface of the scaffold and are, without difficulty, removed all through the cellular tradition at every medium alternate; (ii) precise immobilization, meaning the liposomes are attached covalently to the scaffold’s surface, thus increasing their stabilization [61].

#### 1.2.7. Combining Scaffolds with Exosomes

These days, studies have shown that specific exosomes exist within the body’s circulation and are the key to using force for restoration in damage settings. An increasing number of exosomes, depending on their content material, could induce activation, propagation, differentiation, or apoptosis of the receptor cells. They have the capacity to be both indicators of disease initiation, development, and drug resistance, and a prospective new remedy approach. In recent studies, useful regenerative exosomes are based on different methods to regenerate injured tissue in conjunction with current and experimental bioscaffolds such as collagen. This study showed that an engineering scaffold with CD63 as a molecular design introduces a cell-targeting mechanism to the exosome surface, and allows sturdy and versatile surface engineering of the exosome [129].

## 2. The Application of Musculoskeletal Tissue Engineering Scaffolds Modified with Liposomes or Exosomes

### 2.1. Regeneration of Bone with Exosomes

In contrast to many various tissues, bone tissue has the capacity to improve and reshape [130,131]. Bone tissue engineering in conditions such as bone disease repair has long been a concern for patients and physicians. The latter relies heavily on bone grafting in traditional medical treatments for bone deficiency. [132]. As a significantly greater practical and stable therapy method for bone regeneration, BTE, which includes scaffolds, biological materials, and cell types with osteogenic ability, has been particularly modified to principally solve these issues [133]. This particular research aimed to, investigate the impact of mineral-doped PLA-based porous scaffolds enriched with exosomes on the osteogenic engagement of human adipose mesenchymal stem cells (hAD-MSCs). The involvement of the mineral admixtures improved hAD-MSCs’ osteogenic dedication. Exosomes had been effortlessly applied to the level of the scaffolds. They expanded the expression of genes of primary markers of osteogenesis, which include collagen kind I, osteopontin, osteonectin, and osteocalcin [134]. In a study, it was demonstrated that exosomes were, in most cases, liberated through exosome-coated silk fibroin scaffolds to induce bone marrow mesenchymal (Exo- SF-BMSC Scaffolds) significantly enhancing the process of curing bone. Three-dimensional bioprinting technique scaffolds, entailing of silk fibroin (SF) isolated from mesenchymal stem cells and segregated from predominantly human adipose (hAMSCs), effectively improve bone formation [135].

### 2.2. Regeneration of Bones with Liposomes

**Figure 5:**
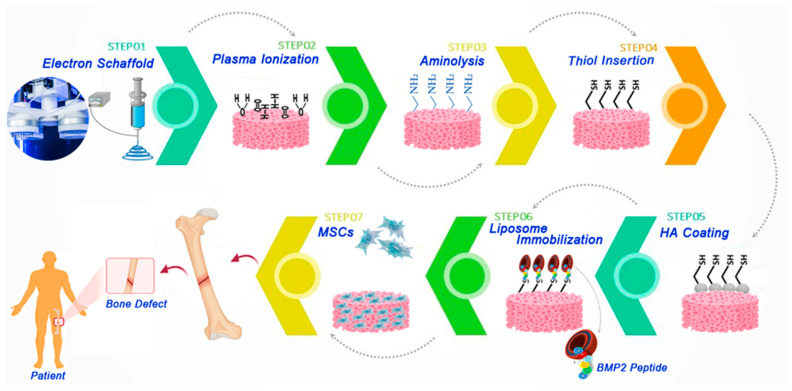
Schematic of the brief representation including conjugation of scaffolds platform, as well as of bone morphogenetic protein−2 peptide-loaded liposomes for fabrication of unique scaffolds for bone fracture regeneration.

Principally, most cytokine forms are involved in controlling the bone regeneration process and activating osteogenic precursor cells, such as osteoblasts and osteoclasts. Firstly, the cells are activated within the procedure of bone absorption; afterward, preosteoblast reproduction may be found beneath the impact of various cytokines [136]. Composite standard scaffolds will liberate the drug in vivo for the duration and occupy the bone imperfection region, which enables specific mechanical protection in cell reproduction, adherence, and deficiency [137]. As shown in Figure 5, favorable biocompatibility, and an exceptional capability to promote osteogenic MSC distinction, may be located within the scaffold. In that same analysis, poly PLA nanofibers imitated the nanofibre design of bone proteins, allowing hydroxyapatite nanoparticles to cover these fibers, and bone morphogenetic protein-2 peptide-packed liposomes, to be implanted on the scaffolds through peptide bonds [138]. In another study, hydroxycholesterol was shown to have the functionality to promoting fundamental bone formation, so in this reaserch 20(S)-hydroxycholesterol was utilized in liposomes as a combination [139]. In the recent project, typically produced DFO-loaded liposomes with elementally modified gelatin methacryloyl (Gelma), substantially released the drug in situ, facilitating angiogenesis and bone regeneration [140].

### 2.3. Cartilage Regeneration with Exosomes

Cartilage is a supple tissue made up of hyaluronic acid, collagen fibers, proteoglycan, and chondrocytes, which also has a restricted ability to regenerate itself after damage or wounds [141]. Due to restrictions in the trade-off to be had in signaling molecules, the ample of oxygen and nutrients, and penetration to progenitor cells; common avascular structural traits, frequently restrict the successful restoration of injured cartilaginous tissues. Trauma, non-stop load-bearing, and various joints problems, particularly osteoarthritis (OA) and rheumatoid arthritis, are the most common causes of articular cartilage disorders [142]. Physicians, on the whole, have long struggled with repairing joint cartilage defects. After an injury, joint cartilage’s capacity to self-heal is severely reduced. If articular cartilage fracture was never completely repaired, this could rapidly lead to osteoarthritis (OA), an extreme bodily and predominantly psychological health hazard [143,144]. One study found that symmetrically guided channels can be used to produce, design, and fabricate exosome 3D printed scaffolds for cartilage repair in tissue engineering. They discovered that the 3D printed scaffold might successfully maintain exosomes in vitro for 14 days, and in vivo for at least seven days [145]. Using a rat knee joint osteochondral defect model, researchers discovered that hWJMSC-Exos could control the anterior cavity’s microenvironment. Furthermore, microRNA (miRNA) sequencing revealed that hWJMSC-Exos contains a large number of miRNAs that facilitate the regeneration of hyaline cartilage [146]. A recent study found that by combining stem cell-derived exosomes into tissue adhesive hydrogels, they could create something similar to a situ-shaped hydrogel matrix as a tissue patch for the sensitive EHG for cartilage fracture reconstruction. EHG tissue patches have been found to blend smoothly with indigenous cartilage and successfully maintain exosomes at the defective location. Additionally, it exhibits positive cellular control in vivo and in vitro, resulting in cartilage reconstruction and restoration [147].

### 2.4. Treating Osteoporosis with Exosomes

The effect of a disbalance between osteoblasts and osteoclasts leads to osteoporosis. Osteoblasts chiefly bring calcium into the bones, reinforcing them, and osteoclasts extract calcium from the bones, leaving them effectively fragile and faint [148]. In all osteoporotic cases, exceeding the osteoclast state causes low bone density; on the other hand, osteoclasts may modulate osteoblasts’ behavior via cytokinetic secretion [149,150]. The objective of the current research was to assess if and how MHA (magnetic hydroxyapatite scaffold) scaffolds encourage osteoblast behavior in the osteoporotic environment through osteoclast-derived exosomes. Amassing evidence shows that, through osteoclast-mediated indirect modulation, magnetic pressure can also promote osteoblast activity [151]. Subsequently, increasing research has verified osteoclasts might also use exosomes to supply proteins, biologically active lipids, and normally genetic correctives into osteoblasts, thereby moderating the viability and even essential organic specifications of osteoblasts [152].

## 3. The Application of Craniomaxillofacial Tissue Engineering Scaffolds Modified with Liposomes or Exosomes

### Craniofacial Bone Regeneration with Exosomes

Today, the use of tissue engineering for bone regeneration is particularly crucial for the craniofacial area. The craniofacial zone is a genuinely complicated tissue, along with bone, cartilage, relatively smooth tissue, nerves, and blood vessels. Various factors may harm these components, such as cancer surgery and congenital abnormalities, which can endanger the functionality and flexibility of bones of the cranial area [153,154]. Cell-free bone tissue engineering approaches containing the biological activities of stem/progenitor cells, to trigger healing and tissue regeneration exceptionally, are also beneficial [155,156]. Exosomes derived primarily from human dental pulp stem cells (hDPSCs) have a pro-osteogenic potential in bone marrow stromal cells (BMSCs). A study of transmission electron microscopy indicated that exosomes predominantly protect their morphology during release. Exosomes obtained from human cells have been shown to be capable of modulating the recipient cell phenotype in a similar fashion, in both human beings and mice, which agrees with current studies suggesting exosome cross-species potency. A synthetic polymer transfer mechanism was built using glycolic acid and lactic acid (PLGA), as well as PEG or polyethylene glycol microspheres of a triblock copolymer, to aid distribution and targeted transfer of osteogenic potential hDPSC-derived (human dental pulp stem cell) exosomes, which promote bone regeneration of bone marrow stromal cells (BMSCs) and contribute to mineralization. This showed that mineralizing exosomes (OS-EXOs) from hDP-SCs could improve MSC osteogenic formation in in vitro and in vivo bone healing [157].

## 4. The Application of Skin Tissue Engineering Scaffolds Modified with Liposomes or Exosomes

### Treatment Diseases Associated with the Skin with Liposomes

The skin includes the epidermis, dermis, and hypodermis tissue, which is the largest organ in the human body. The skin is made up of keratin, a vital protein that avoids dehydration, and also protects the tissues, organs, and components under the skin from immediate environmental harm, such as pressure and temperature. It can also shield a range of specific organs and kinds of tissue in the body from the influx of pathogenic microorganisms [158]. Some studies aim to develop liposome-scaffold composite systems according to the skin characteristics and each layer’s functions, which may satisfy particular clinical requirements. Some principally phenolic compounds, such as nailbed matrix ablation, are routinely utilized to cure skin disorders. As this possesses analgesic, anti-inflammatory, and anti-allergic properties, as well as antipyretics activity, most phenolic compounds can easily penetrate the skin through absorption [159,160]. In new medical research, Xia et al. developed liposome loading by paeonol, which has been mixed with these liposomes using hydrogel to improve the localized retention period and skin adhesion to reach a higher cure effect [161].

## 5. The Application of Neural Tissue Engineering Scaffolds Modified with Liposomes or Exosomes

### 5.1. Reparation of the Spinal Cord with Liposome

Permanent functional illness may result from spinal wire injury, together with neuronal and axonal damage; moreover, currently, neural stem cells (NSCs) have progressively seemed likely cell resources for spinal twine regeneration [162,163]. The structural scaffolds may provide an efficient micro-environment for NSCs to turn into mature neurons and viable sensory and motor neurons, and an efficient micro-environment for neural stem cell proliferation, motor neuron regeneration, and sensory neurons. According to studies, the microtubule-stabilizing agent paclitaxel (MSPTX) may decrease scar production and increase the internal axon regeneration after spinal cord damage. For this reason, a compound collagen microwell scaffold, comprising MSPTX-loaded liposomes and primarily neural stem cells, was constructed. The collagen scaffolds with PTX functionalization may reduce myelin inhibition, and enhance NSCs’ intrinsic neuronal differentiation capacity in vitro [164].

### 5.2. Spinal Cord Injury Treatment with Exosomes

Spinal cord harm (SCI) is an excessive neurological trauma with high morbidity and mortality, which can result in permanent incapacity due to a lack of sensorimotor characteristics under the lesion [165]. For SCI treatment, scaffold characteristics of non-toxic and non-carcinogenic features, biocompatibility, and biodegradability are essential [166]. Scaffolds have the most effective bioadaptability and little immunogenicity, and are therefore in a position to build a favorable SCI micro-environment [167]. In addition to transporter cells and biologically active factors, bioscaffolds may reconnect lesion holes, which could, in turn, enhance axonal and functional restoration. Axonal regulation is an essential aspect of nerve recovery and, due to inhibition roles in their plasma membrane, axonal restoration of the CNS in its natural area is complex [168]. The loading of specified materials that also have anti-inflammatory outcomes may enhance SCI. As certainly one element of SCI pathogenesis is the incitement of signaling routes by using inflammatory elements, EVs ought to be used to suppress the potentially harmful inflammatory flow. The periphery flow stabilization of EVs may pass their components to the host cell, and might even be combined with the scaffold or stem cells to aid in neural growth and create a favorable SCI recovery micro-environment [169].

### 5.3. Exosomes as a Traumatic Brain Injury (TBI) Treatment

TBI, which would be induced by a hit or shake on the head, has become a life-threatening disorder that is the leading risk factor for death and principally lengthy disabilities globally [170]. The primary causes of mortality in TBI instances are increasing in number including car crashes and violence, armed conflicts, or terrorist acts accounting for the majority of cases. Adolescents, mostly males, make up the majority of TBI cases. The brain’s complexities and the presence of the blood–brain barrier (BBB) make it difficult to implement successful modern treatments via plain venous injection [171]. Clinical studies in TBI have not yielded any successful pharmacokinetic therapies to date. As a result, throughout the occurrence of traumatic brain injury (TBI) and other brain damage, an appropriate clinical distribution system is required [172]. TBI cancer could be improved by singularly combining exosomes, bioscaffolds, and stem cells. Exosomes are glycoproteins containing mannose that play a role in neuron-to-neuron interaction, regulated by special glycosylated sialic acid and myelinated oligodendrocytes. They serve as biological markers for cancer therapy [173] according to the latest review, exosomes extracted via MSCs in 2D culture improved mental performance and reduced inflammation in TBI mice, which obtained 30 g of exosomes one hour after injury [174] according to a current survey, hMSC-generated exosomes appear to dramatically boost wound healing in rats following TBI, predominantly through encouraging endogenous angiogenesis and neurogenesis, and reducing neuroinflammation. Exosomes extracted through hMSCs could perhaps become an innovative, almost always cell-free treatment for TBI. These exosomes, produced by an hMSC scaffold, can generally improve spatial learning systematically [175].

### 5.4. Therapy for Head Injuries with Exosomes

Traumatic brain injury (TBI) is also a severe disorder that could result in incapacity or even death. The presence of the blood–brain barrier (BBB), together with the brain’s complexity, complicates the transfer of successful therapies through a plain intra-arterial infusion. Hence, it may help move stem cells to the damaged sites by combining biomaterials with altered exosomes, thus still growing their viability and facilitating successful care. [169,170] Neuroinflammation is also a crucial therapy aim for TBI due to its ability to contribute to secondary harm. Even to avoid TBI damage, exosomes are presumed to modify immunity activity [169]. To cure rats with skin injuries and periphery nerve wounds, an extremely porous collagen scaffold was utilized. Research findings demonstrated that the scaffold substantially prevented glia scar-forming and injury contracting, and facilitated nerve reconstruction [176].

## 6. The Application of Dental Tissue Engineering Scaffolds Modified with Liposomes or Exosomes

### Regeneration of Teeth

The existence of dental restorations is, in most cases, presently very limited. However, the use of usually regenerative medicine to primarily facilitate the dentin-pulp complex’s regeneration means an exceptionally great deal to the profitable industry of restorative dentistry. A variety of definite bioactive agents and principally outer membrane proteins are substantially associated with the absorption, differentiation, and duplication of dentine matrix pulp generating cells. The potential capacity of manufactured decalcified tooth matrix loaded liposomes (DDM-Lip) to promote in vitro teeth genesis, an undoubtedly unique technique for teeth restoration and rigid tissue engineering, has been notably anddistinctly demonstrated in one study. Findings show DPSC is a required agent in the true reproductive function of teeth textures. Peculiarly, more proficiency in absorption and activating dental pulp stem cell (DPSC) was observed in DDM-Lip remedied models compared to the DDM remedied model [60,177].

## 7. The Application of Scaffolds for Reproductive System Modified with Liposomes or Exosomes

### Female Fertility Preservation with Liposomes

On the whole, the female endometrial lining is a highly dynamic reusable tissue that endures approximately four hundred menstrual cycles and can receive embryos through implantation during a female’s reproductive period [178]. Trauma prompted by repeated curettage, cesarean phase, myomectomy, or infection, frequently results in uterine adhesions (IUAs) and infertility. In a study, forty-three percent of females (802 out of 2151) were confirmed to have infertility with IUAs; extreme IUAs, due to excessive fibrosis and thinner endometrial lining, often lead to infertility [179,180]. Recently, the improvement of uterine endometrial health in rats has been investigated with the structure of exosomes and collagen scaffolds (CS/Exos). Specific CS/Exos regeneration allowed endometrial reconstruction; collagen remodeling enhanced ER-alpha and PR expression throughout the revived endometrium and remarkably improved fertility recovery [181]. A principal medical study has shown that combining CS with MSCs into the uterus cavity in female IUA cases will facilitate endometrial regeneration [182]. In the rat IUA model’s reproductive investigation, MSC-derived exosomes (25 μg) were handled by injection in uterus horns to promote endometrial restoration [183].

## 8. The Usage of Scaffolds Composed with Liposomes in Sickness

### 8.1. Breast Cancer

Breast cancer has been one of the more frequent malignancies among females, responsible for approximately 30% of all cancers and 15% of all cancer deaths in women [184]. Due to its rapid invasion and relapse characteristics, a powerful and accurate remedy for breast cancer is desperately required [185]. Surgery accompanied by chemotherapy seems to be the current standard care technique [186]. However, it seems the substantially surgical procedure cannot eradicate cancerous tissue. Insolubility in water and toxicity, particularly in natural granulation tissue, often seriously restricts the use of many of these chemotherapy drugs [187]. In general, positional drug delivery in the carcinoma region is a much more effective and less toxic treatment for tumor cells than conventional chemotherapy with the aid of injection.

On the other hand, the choice of an effective drug carrier and the managed secretion of chemotherapy drugs remain significant challenges. These distinctions might not have had the most significant impact on cancer tissue; however, they would benefit normal tissue. Chemotherapeutic drugs predominantly affect normal tissues rather than cancer tissue [188]. Generally, uncontrolled drug release can result in cancer tissues resisting therapy, while the surrounding cells are substantially damaged. On the other hand, the complicated surroundings in vivo can distinctly lead to drug decomposition [189,190]. A hybrid drug carrier device incorporating nano-drug carriers, and principally implanting material, has been suggested to resolve these issues, which would induce a limited immune response reaction while causing no harm to tissues and organs [191,192]. A recent study used paclitaxel (PTX), loaded with liposomes composed of phospholipids and waterborne polyurethane (WBPU), to create an engineered, dual-encapsulated, and biodegradable 3D bioprinting scaffold for controlling local drug release in the treatment of breast cancer. The cell test findings show that the dual-encapsulated scaffold inhibits breast cancer mcf7 cells more effectively, and causes reduced toxicity to adjacent tissue cells. Furthermore, this dual-encapsulated biodegradable 3D scaffold can broadly prevent tumor growth, while effectively encouraging substantial natural cell growth [193].

### 8.2. Cancer Treatment

In cancer-affected persons, capillaries’ penetrance ability in the lesion is extended, due to the irritation and contamination triggered with the aid of the stable tumor formation. Some liposomes may display successful cancer-targeting potential, or carry drugs to targeted organs, cells, or subcellular organelles. Such liposome specification allows them to be assigned to cancer remedies to design compound liposome scaffolds [60]. Mao and colleagues created novel temperature-responsive injected novel hydrogel, comprising liposomes loaded by paclitaxel(PTX), in 2016; after the preparation, the transformation temperature and rate of drug delivery were surveyed in vitro [194]. Furthermore, in another study, Xing et al.’s in vivo studies showed that these liposomes could be transferred to the tumor’s local region, increasing the drug’s chemotherapy and bioavailability effectiveness [195].

### 8.3. Diabetes Mellitus

Diabetes is one of the most famous metabolic sicknesses globally, in which the insulin hormone is dysregulated. Indeed, glucose remains in your blood without adequate insulin. Two primary forms of diabetes are type one and type two. In type one diabetes, your body no longer releases insulin. The more significantly extreme type, type two diabetes, is characterized by the body being unable to make or use insulin optimally [196]. A heat-sensitive hydrogel, modified with liposomes storing insulin, was created to shorten infusion time and prevent side effects, such as pain from several infusions and infections from exposing an insulin pump to the catheterization region [197]. Additionally, a form of the hydrogel capable of being injected, comprising separated islets of langerhans and the clodronate-containing liposomes, was initially developed to treat type 1 diabetes effectively. The above hydrogel was subsequently utilized to treat SD rats lacking islets of langerhans, which resulted in a considerable increase in SD animals’ average survival period of 2 months [198].

### 8.4. Inflammatory Disorders

The immunity system’s reaction to a damaging stimulus, such as pathogens, destroy tissue (broken cells), poisonous compounds, or radioactive chemicals, is inflammation. Inflammation performs an essential position in recovery but also risks worsening the danger of several diseases, along with some cancers and rheumatoid arthritis, which can be exacerbated by chronic inflammation [199]. Redness, swelling, fever, ache, and dysfunction are among the most critical signs of inflammation. When irritation and infection occur, chemical substances from our white blood cells penetrate our bloodstream and organs to protect us against invaders [200]. However, in the inflammatory response proceeding, scaffolds composed of liposomes are most often applied for anti-inflammatory treatment. For instance, engineered resveratrol loading liposomes were further processed with just a hydrogel by benefiting from both ethanol infusion and film scattering methods. This method should substantially extend the therapy period and decrease the degree of in vitro swelling in contrast to the well-known diclofenac sodium gel [201].

### 8.5. The Human Immunodeficiency Viruses (HIV)

HIV is a virus that targets the immune system comprised of two kinds of lentivirus (a retrovirus subgroup) that each contain a duplicate copy of the single-stranded RNA genome. They generate the immunodeficiency syndrome (AIDS), a disease in which, unless treated, life-threatening infections and typically lethal tumors primarily characterize innovative immune device failure [202]. A composite method for HIV treatment in the vaginal mucosa was created with the help of a hydrogel matrix to contain (MVC+TDF), Maraviroc (MVC) mixed with tenofovir disoproxil fumarate (TDF), resulting in HIV-1 bal antiviral attempt in the cervicovaginal [203]. In another study about dual HIV inhibitors, new C12H6O5 (4-Hydroxy-2H,5H-pyrano[3,2-c][1]benzopyran-2,5-dione) formatives are being synthesized to manipulate a coumarin-based scaffold to attain the prohibition of more than one viral coded enzymatic roles [204].

### 8.6. Anti-Bacterial Activities and Applications

A bacterial infection is a propagation of a dangerous type of bacteria within the body. It may present specific general prevalent symptoms, such as discomfort, lasting fever, headache, nausea, and swollen lymph nodes [205]. In the latest discoveries in the anti-bacterial application, chitosan-based hydrogels have been coupled to liposomes containing mupirocin to complete a newly designed hydrogel mechanism, enhancing wound restoration proven through the transport mechanism’s findings [206]. The scaffold was related to liposomal gentamicin (GS) in another research to design an innovative Porous β-tricalcium phosphate (β-TCP) which allowed a different route for the regulated distribution of liposomal antibiotics, to remedy osteomyelitis induced through continuing bacterial infection [207].

### 8.7. Healing of Acute Wounds

Wound healing appears to be a multi-step process involving the regeneration of various tissues (including epidermal cells), the development of periodontal tissue proliferation, and the formation of granulation tissue. Inflammation, infection, and anti-infection agents all play essential roles in wound healing [140]. The abundance of FGF (fibroblast growth factors) on the wound area, together with a significant sore leachate volume, is a critical feature in the wound therapy strategy. To prove this, a study took advantage of the modification of liposome hydrogel with polyvinyl-pyrrolidone iodine (PVP-I) to remedy those affected with second-degree scald wounds. The findings revealed that there existed another water-binding level inside the compound hydrogel matrix, which might provide crucial moisture towards the defect zone. Additionally, FGF performs a fundamental wound healing routine; it may also speed up novel blood vessel development and restore broken endothelial cells [208]. In addition, specific growth factors of fibroblast (bFGF) loaded-silk fibroin hydrogel had been integrated into the internal phase of liposomes for the transporter, to distribute growth factors to the burn wound region [209]. Mupirocin was manufactured with chitosan hydrogel liposomes to reduce the venture of wound infection, resulting in a significant inhibitory effect on staphylococcus aureus and bacillus subtilis [210].

## 9. The Usage of Scaffolds Composed with Exosomes in Sickness

### 9.1. Wound Healing

The primary problems in the remedy of principal skin tissue injuries are scar creation and lag wound healing. Wound healing is one of the most sophisticated biological methods for repairing skin injured by surgery, wounds, burns, or, most notably, diabetic diseases resulting in a deficient matrix of fibrous skin [211]. In pathologically severe situations, such as injury, burns, and diabetic wounds, natural aggregation and substantially native dermal healing mechanisms will not be able to repair damages [212]. Skin replacements have recently emerged as a promising medicinal alternative for the treatment of most cases of skin wounds, due to the use of biocompatible scaffolds in the presence of stem cells and biologically active substances. In order to have good curing properties, an optimal skin replacement must usually have substantial porosity and permeance [213]. Consequently, fibroblasts could recognize and absorb differentiated EXOs from adipose-derived stem cells (ADSCs) and alter their activities, which included migration, replication, and collagen synthesis [214]. Furthermore, one possible function of ADSC-derived EXOs’ wound-healing capacity may effectively be an improvement in fibroblasts’ potential in the generally healthy skin to migrate, which may aid wound contraction [215]. In a recent study, researchers examined whether ADSC-derived EXO transmitted via an Alg-based hydrogel could help cure a dermal percutaneous wound pattern. The findings showed that the manufactured structure was mostly environmentally friendly and demonstrated excellent biocompatibility. Altogether, wound healing, collagen formation, and distinct vessel construction in the injury region have increased dramatically according to this biologically active wound treatment procedure [216].

### 9.2. Engineering Smart Exosome–Liposome Hybrid

As liposomes and exosomes have many elemental similarities, using engineering technologies to create a, for all intents and purposes, hybrid exosome-liposome has been suggested to resolve their restrictions. Many of these comparisons have demonstrated that exosomes, especially, have benefits over liposomes and vice versa [105]. On the other hand, liposomes need to have their surfaces substantially modified with ligands in order to gain significant, singularly clever, and broadly aiming capabilities. The above characteristics are typically already present in certain natural nanovesicles, such as exosomes. Donation cells substantially provide exosomes with intelligent action in the shape of cellular and lipid adherence molecules [217]. Even so, with the use of the methods and techniques developed in the field of liposome engineering, researchers may be able to design exosomes to load drugs into them or bind specific molecules to their level [102].

**Figure 6:**
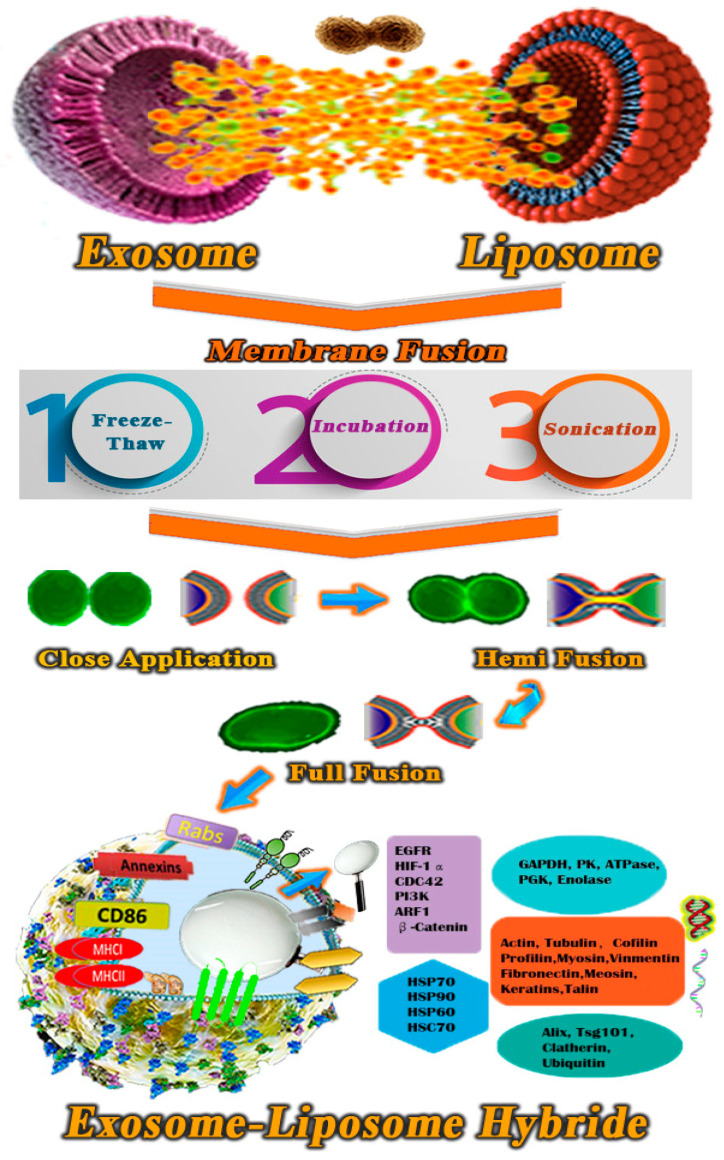
The procedure for creating exosome–liposome hybrids is depicted in a formation. Sonication, incubation, and freeze–thaw cycles were used as procedures.

According to the hybrid exosomes’ cellular defense experiments, the exosome distribution mechanism may be altered by modifying the lipid structure or characteristics of the exosomes by membrane fusion. These findings suggest that adding exogenous lipids to hybrid exosomes will, on the whole, alter exosome–cell connections [218]. Exosomes that have usually been genetically or non-genetically engineered may improve the cytotoxic effects and the whole targeting capacity of therapeutic agents, thus increasing their drug delivery efficacy [102]. Engineering hybrid exosome-liposomes by means of manipulating exosomes and fusing their membranes with artificial liposomes seeks to create exosome–liposome hybrids with longer half-lives within the bloodstream. Figure 6 illustrates the three principal processes for engineering hybrid exosome-liposome: incubation, freeze–thaw, and sonication [105]. Polyethylene glycol (PEG) on the liposome membrane enhances their blood circulation times, whereas, in most cases, it decreases their absorption via mononuclear phagocytic cells. These results suggest that PEG reduces conflicts between liposomes and cellular surfaces, allowing PEG-modified exosomes to be taken up by cells efficiently [219]. In the most recent study, researchers attempted to encapsulate wide plasmid double-stranded DNA (pDNA) inside exosomes, then deliver it to MSCs (mesenchymal stem cells). In summary, they tried several methods to encapsulate the CRISPR–Cas9 technology into extracellular vesicles, but found that the suggested engineering hybrid exosome fused with liposome incubation could become a unique technique for medication encapsulation and trying to deliver the CRISPR–Cas9 in in vivo and in vitro models. The exosome–liposome hybrid nanoparticles, when combined, may carry the CRISPR–Cas9 system to MSCs, singularly making them useful for in vivo genetic manipulating [108].

## 10. Critical Discussion and Perspective

Despite many advancements in regenerative medicine, there remain several obstacles related to cellular, scaffolds, and signaling. In addition, as each type of stem cell has its own set of benefits and drawbacks (immune system response, maturation, etc.), determining the best resource for such cell types and subsequent cultivation is a difficult task within itself [220]. Hence, choosing biomaterial for scaffolds is not simple. Nevertheless, the scaffolds should effectively adapt to both the bodies’ virtually natural structure and functioning needs. Furthermore, this must have biocompatibility and the capability to communicate with the ECM, while also giving mechanical backing [221]. Although biological substances seem to be more biocompatible and biodegradable, synthetic materials often possess more potent mechanical characteristics. This is why composite materials are occasionally necessary, as they permit the scaffolds to maintain their porosity composition [222]. An further significant problem for tissue engineering involves nutrition and waste secretion conveyance in principally created tissues [223].

The three-dimensional manufactured tissues must be connected to the bloodstream with a vascular system, as many cells rely on the vascular system to deliver oxygen and nutrition [224]. However, this is not a simple process; when the scaffolds are, in most cases, implanted within the body, all existing oxygen is immediately exhausted, while new capillaries are predominantly created after just a few days [220]. As a result, new methods of angiogenesis are required, and several methodologies for the creation of synthetic organ revascularization have been proposed, utilizing generative, incremental, and combination techniques [225]. Furthermore, as the size of the manufactured tissue grows larger, feeding and oxygen delivery to preserve cellular viability and promote cell growth becomes increasingly difficult, necessitating adequate tissue vascularization. Dynamical culturing inside bioreactors might be a way to speed up the development of avascular tissues and extend their survival [226]. However, exosome detection methods, including a Western blot (WB) and flow cytometry (FC), are time-consuming and confusing. Flow cytometry can now analyze nanoparticles as tiny as 30 nm, but it cannot accurately detect exosomes, as it relies on standard particles for enumeration, decreasing its sensitivity [227]. Therefore, mesenchymal stem cells (MSCs) are considered the most effective potential options for exosome formation from a medicinal viewpoint. Indeed MSC-derived exosomes have had the capacity to modulate the immunological system and the potential to move towards the inflammation area, making them attractive for therapeutic applications. Exosomes produced from MSCs also have the potential to communicate within cells [227,228].

Moreover, exosomes contain the kind of unique biomarkers from their mother cells which might, on the whole, be utilized for diagnostics, as well as treatment applications. However, to fully understand the possibilities of exosomes as cancer treatments, more research is needed. Exosomes generated by stem cells have also been effectively utilized to promote osteogenesis in vivo, allowing for both development of new blood vessels and the formation of bone [227,229].

On the other hand, liposomes primarily have three major disadvantages that severely restrict their therapeutic use. First, liposomes may not withstand shear pressures or changes in temperature, diluents, pH value, or diluent concentrations. Second, liposomes are extremely sensitive to external stimuli and their responses, which precludes broad use in drug delivery. Third, delivering compounds within liposomes to the particular location in vivo with precision is difficult [230].

## 11. Closing Remarks

To sum up, tissue engineering (TE) is among the most extraordinarily fascinating cross-disciplinary and multi-disciplinary researching fields, and it is rapidly expanding. Nevertheless, tissue engineering is still in the early stages of development. The advancement of technological tools and better knowledge of biology and immunology is critical for the field’s future progress. The ultimate objective of any research is, predominantly, to create a particularly functioning organ that can operate following being implanted and interact with neighboring organs and remote tissues. Biological materials for scaffolds and manufacturing processes are critical in tissue engineering and regenerative medicine, and they are rapidly developing. Various fields, including science, treatment, and materials technology, have been integrated in this discipline, and indeed the fundamental aim of tissue regeneration has been achieved. The physical, biochemical, and biomedical characteristics of tissue scaffolds are the essential factors to consider during design and construction. They affect their connection to cells and tissues, biodegradation, and non-toxicity, among other things. In general, 3D bioprinting has already been quickly growing amid current manufacturing techniques due to the unlimited benefits of nanoscale, excellent throughput, and cellular depositing. Although polymeric materials can provide enough mechanical stability and hardness for structures, composite bioceramics are more suitable, due to their inherent biocompatibility, high osteoconductivity, and bioresorbable. In successful scaffold-based tissue engineering, a nimble biological material replicates the composite structures of the extracellular matrix (ECM). It then engages with organelles in a dynamical fashion that promotes adherence, proliferating, differentiating, and cellular formation. Chiefly, artificial intelligence (AI) and big data analytics (BDA) would dramatically speed up the planning, manufacture, and evaluation of TE technology processes, improve clinical care, and save money in the societal and medicine sectors.

Exosomes are extracellular vesicles (EVs) as messengers of intercellular that carry cargoes and purposes, including DNA, RNA, and proteins, from mother cells to the targeted cells, altering their operational status. Even though the research of exosome applications has progressed significantly, numerous challenges remain in specific sectors: (1) exosome centrifuge and industrial kits are now effectively unable to extract exosomes precisely and comprehensively. As a result, the process must be more precise, standard, quick, inexpensive, simple, and particular in terms of extraction and filtration procedures and fluid biopsies technologies. (2) Exosome manufacturing on a massive scale should principally be, in most cases, investigated for clinical use. (3) To ensure efficient operation, or even minimize side effects, more study of exosomes is required. (4) To use exosomes in clinical action, much in vivo research is required. In addition, more research on the effectiveness and toxicology of the product is needed. Finally, (5) the exact dose of exosomes as a carrier and their medicines is unknown. As illustrated in this study, exosomes have the potential to become a new generation of unique nanocarriers for drug delivery.

Liposomes are beyond doubt one of the most exceptionally strong families in the field of biomedical sciences. The following are the key factors contributing to the poor liposome transference rates between bench to bedside: inherent cytotoxic capability, leakage rate, stability issues, batches to batches reproductions, and effective sterilizing techniques. In addition, liposomes have several benefits as drug delivery systems, including lymphatic structure tropism, passively focusing on bone marrow and spleen, significant regulation of release of drugs, reducing medication side effects, and improved drug persistence. On the other hand, liposomes confront substantial challenges, such as storing and delivering medicines. Furthermore, liposomes have difficulty maintaining medication dosages in positional regions for lengthy durations of time. As a result, those drawbacks must be fundamentally addressed, without significantly jeopardizing the benefits of liposomes in therapeutic usages. Concocting composite scaffolds using liposomes allows scaffolds to promulgate, regenerate, and cure illnesses, and allows the medication concentrations to maintained in situ for an extended length of time.

Furthermore, scaffolds engineered by liposomes distinctly demonstrated long-term medication delivery. Additionally, homogeneous liposome solutions containing various medicines that can be utilized to deliver pharmaceuticals throughout the scaffold equally may be readily created. Therefore, bioengineered liposome-scaffold composites can transport a wide range of medications using this approach, allowing for synergy treatment. Nevertheless, currently, a predominantly specific issue with the composite system is that it would almost be impossible to determine and provide the precise amount to both the patient or animal models whenever the medicine is administered this way.

## Figures and Tables

**Table 1:** Structural Classification of Liposomes Based on Size and Number of Lamellae.

Lamellarity	Abbreviation	Number of Lipid Bilayers	Diameter Size Rang Structures	
**(1) Unilamellar Vesicles**	**ULV**	**one lipid bilayer**	**All size range**	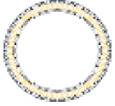
**Dimensions**				
**Name**	**Abbreviation**	**Size Range (µm)**	**Characteristics**	
Single Unilamellar Vesicles	SUV	0.02–0.20	useful for entrapping lipophilic active materials	
Medium Unilamellar Vesicles	MUV	0.20–0.50	slightly more efficient than SUV	
Large Unilamellar Vesicles	LUV	0.50–10	capable of capturing a significant amount of hydrophilic material	
Giant Unilamellar Vesicles	GUV	100–200	look like cell membranes, ideal templates for microscale bioreactors	
**(2) Oligolamellar Vesicles**	**OLV**	**few concentric lipid bilayers**	**100–500 nm**	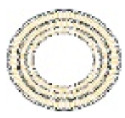
**(3) Multilamellar Vesicles**	**MLV**	**many concentric lipid bilayers**	**>500 nm**	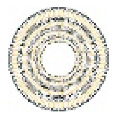
**(4) Multivesicular Vesicles**	**MVV**	**non-concentric vesicles within a single lipid bilayer**	**>1000 nm**	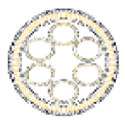

## Data Availability

The data presented in this study are available on request from the corresponding author.
